# Carboxypeptidase G_2_ rescue in patients with methotrexate intoxication and renal failure

**DOI:** 10.1038/sj.bjc.6602337

**Published:** 2005-01-25

**Authors:** S Buchen, D Ngampolo, R G Melton, C Hasan, A Zoubek, G Henze, U Bode, G Fleischhack

**Affiliations:** 1 Department of Paediatric Haematology/Oncology, Children's Medical Hospital, University of Bonn, Bonn, Germany; 2 Protherics Plc, Salisbury, UK; 3 St Anna Children's Hospital, Vienna, Austria; 4 Department of Paediatric Haematology/Oncology, Charité, Humboldt University of Berlin, Germany

**Keywords:** high-dose methotrexate, acute renal failure, methotrexate toxicity, carboxypeptidase G_2_

## Abstract

The methotrexate (MTX) rescue agent carboxypeptidase G_2_ (CPDG_2_) rapidly hydrolyses MTX to the inactive metabolite DAMPA (4-[[2,4-diamino-6-(pteridinyl)methyl]-methylamino]-benzoic acid) and glutamate in patients with MTX-induced renal failure and delayed MTX excretion. DAMPA is thought to be an inactive metabolite of MTX because it is not an effective inhibitor of the MTX target enzyme dihydrofolate reductase. DAMPA is eliminated more rapidly than MTX in these patients, which suggests a nonrenal route of elimination. In a phase II study (May 1997–March 2002), CPDG_2_ was administered intravenously to 82 patients at a median dose of 50 U kg^−1^ (range 33–60 U kg^−1^). Eligible patients for this study had serum MTX concentrations of >10 *μ*M at 36 h or >5 *μ*M at 42 h after start of MTX infusion and documented renal failure (serum creatinine ⩾1.5 times the upper limit of normal). Immediately before CPDG_2_ administration, a median MTX serum level of 11.93 *μ*M (range 0.52–901 *μ*M) was documented. Carboxypeptidase G_2_ was given at a median of 52 h (range 25–178 h) following the start of an MTX infusion of 1–12 g m^−2^ 4–36 h^−1^ and resulted in a rapid 97% (range 73–99%) reduction of the MTX serum level. Toxicity related to CPDG_2_ was not observed. Toxicity related to MTX was documented in about half the patients; four patients died despite CPDG_2_ administration due to severe myelosuppression and septic complications. In conclusion, administration of CPDG_2_ is a well-tolerated, safe and a very effective way of MTX elimination in delayed excretion due to renal failure.

High-dose methotrexate (HD-MTX) of more than 1 g m^−2^ body surface area, administered by a prolonged intravenous infusion with leucovorin (LV, D,L-5-formyltetrahydrofolic acid) rescue, has demonstrated clinical activity in malignancies and diseases, such as acute lymphoblastic leukaemia, lymphoma, osteosarcoma, and head and neck cancer.

Methotrexate is primarily excreted by the renal route. It may cause acute nephrotoxicity, which is presumed to result from its precipitation or its relatively insoluble metabolites in acidic urine. This nephrotoxicity leads to delayed MTX elimination, ineffective rescue and a marked increase of other nonhaematological and haematological toxicities associated with MTX, such as myelosuppression, oro-gastrointestinal mucositis and dermatitis. In early studies using HD-MTX, severe toxicity occurred in approximately 10% of patients and there was a 6% mortality rate (Ahmad *et al*, 1978). The introduction of pretreatment hydration, alkalinisation of urine, routine monitoring of serum MTX concentrations and postinfusion guided LV rescue has decreased the incidence of severe and life-threatening toxicity after HD-MTX from 10% to less than 1% (Jüergens *et al*, 1983; Allegra, 1990). However, severe nephrotoxicity of HD-MTX leading to potentially lethal toxicity still occurs.

For patients with decreased MTX clearance due to renal dysfunction, therapeutic options are few and of limited efficacy. When serum MTX concentrations are persistently greater than 10–100 *μ*M, high doses of LV are not likely to completely reverse the toxicity of MTX (Goldman and Levy, 1968; Pinedo *et al*, 1976; Frei *et al*, 1980; Grem *et al*, 1991).

Thymidine may be used in case of MTX toxicity, but due to its rapid clearance it must be administered in high doses by continuous infusion (Ensminger and Frei, 1977; Howell *et al*, 1978; Howell *et al*, 1980; van den Bongard *et al*, 2001). Neither LV nor thymidine has any effect on the underlying problem of delayed MTX elimination. Peritoneal dialysis is ineffective, most likely due to a combination of factors including the protein binding, the high degree of ionisation and the low lipid solubility of MTX (Ahmad *et al*, 1978). Haemodialysis and haemoperfusion have variable efficacy and usually produce only transient decreases of plasma MTX concentrations (Gibson *et al*, 1978; Relling *et al*, 1988; Saland *et al*, 2002). Alternative methods to treat persistently elevated MTX levels are therefore needed.

The carboxypeptidase G class of enzymes hydrolyse the C-terminal glutamate residue from folic acid and classical antifolates, such as MTX, rendering them inactive (Levy and Goldman, 1967; Chabner *et al*, 1972, Goldman, 1975).

Carboxypeptidase G_1_ (CPDG_1_) was originally extracted from *Pseudomonas stutzeri*. During the 1970s, CPDG_1_ was administered successfully to a small number of patients with brain tumours and to one patient with pre-existing renal failure after MTX administration. However, the bacterial source for CPDG_1_ is no longer available (Bertino *et al*, 1974; Abelson *et al*, 1978; Minton *et al*, 1983).

A recombinant form of CPDG_2_, cloned from *Pseudomonas* strain RS-16, is now available as an investigational drug (Minton *et al*, 1983; Sherwood *et al*, 1985). Carboxypeptidase G_2_ hydrolyses MTX rapidly to the inactive metabolites DAMPA (4-[[2,4-diamino-6-(pteridinyl)methyl]-methylamino]-benzoic acid) and glutamate. In contrast to CPDG_1_, CPDG_2_ has a higher affinity for MTX (Km 8 *μ*M) than LV (Km 120 *μ*M) and 5-methyltetrahydrofolate (Km 35 *μ*M), the primary circulating metabolite of LV. In studies performed in rhesus monkeys, a single CPDG_2_ dose of 50 units (U) kg^−1^ lowered serum MTX steady-state concentration from 10 *μ*M to nontoxic levels of <0.05 *μ*M within 30 min (Adamson *et al*, 1992). In addition, the bolus injection of CPDG_2_ (a dose of 50 U kg^−1^ in combination with LV and partial thymidine rescue) resulted in a rapid 95–99% reduction of plasma levels of MTX in patients with renal dysfunction after HD-MTX (Widemann *et al*, 1995, 1997; Zoubek *et al*, 1995).

This study will demonstrate the feasibility, effectiveness and toxicity of an emergency therapy with CPDG_2_ in a large European patient collective with acute MTX intoxication and renal failure.

## PATIENTS AND METHODS

### Patients

#### Study design

The study was a prospective, open, nonrandomised, multicentre trial. The primary study centres were in Bonn, Berlin and Vienna. Their task consisted of consultation and emergency dispatch of CPDG_2_ for patients with MTX intoxication. The substance was requested by 50 different hospitals in 13 different countries (Austria, Belgium, Czechia, France, Germany, Greece, Italy, Israel, Norway, Slovakia, Spain, Sweden and Switzerland).

#### Patient eligibility

Patients of any age were eligible if they had (1) serum MTX concentration of >10 *μ*M at 36 h or of >5 *μ*M at 42 h or of >3 *μ*M at 48 h after start of MTX infusion, and (2) decreased diuresis (less than 50% excretion of the input hydration) or (3) a serum creatinine (Crea_S_) >1.5 times the upper limit of normal and documented increase of Crea_S_ during the infusion period.

### Treatment

#### Carboxypeptidase G_2_ administration

Recombinant CPDG_2_ was manufactured by the Centre for Applied Microbiology and Research (Salisbury, UK) and supplied in lyophilised form with vials containing 1000 U of enzyme activity. The vials were each resuspended in 1 ml of sterile isotonic saline; further dilution with sterile isotonic saline (1 : 5 or 1 : 10) was recommended. Carboxypeptidase G_2_ was administered at a dose of 50 U kg^−1^ over 5 min intravenously by an infusion pump or by bolus injection. Following each dose of CPDG_2_, serum MTX concentrations were determined. Patients who experienced a decrease in serum MTX concentration greater than one logarithm following CPDG_2_ administration but still had serum MTX concentrations >1 *μ*M might receive additional doses of CPDG_2_ with the approval of the principal investigator of the study.

#### Leucovorin

Leucovorin administration had to be stopped 4 h prior to CPDG_2_ and readministered 1 h following enzyme injection. The recommended LV dose during the first 24 h after the CPDG_2_ injection was 100 mg m^−2^ every 6 h followed by an increased rescue for 5 days according to the scheme recommended in the ALL BFM 95 protocol (Schrappe *et al*, 1995).

#### Patient monitoring

Patients were evaluated daily for signs and symptoms of MTX toxicity. Complete blood counts with differential, bilirubin and ALT, AST were determined at least twice weekly. Recovery of renal function was documented daily by serum creatinine. The nonhaematological (i.e. renal, liver, neurological, gastrointestinal toxicity and infectious complications) and haematological toxicity of MTX and side effects of CPDG_2_ (especially allergic reactions) were determined according to National Cancer Institute Common Toxicity Criteria (CTC) version 2.0.

#### Sampling for determination of MTX concentration

EDTA samples for MTX determination were obtained immediately before and 15, 30, 60 and 120 min following the dose of CPDG_2_, and then once daily until recovery of kidney function and decrease of MTX serum levels to less than 0.1 *μ*M. Most of the local hospitals determined the MTX level immediately with the methods such as fluorescence polarisation immunoassay (TDX) or enzyme-multiplied immunotechnique (EMIT). In a few centres, additional blood samples for analysis by high-pressure liquid chromatography (HPLC) were placed on ice, and serum was rapidly separated by centrifugation. To inactivate CPDG_2_, the serum samples were heated to greater than 80°C for 5 min in a water bath or treated with 1 N HCl to give a final concentration of 0.1 N hydrochloric acid. If the enzyme activity in the samples was not fully stopped, MTX in the sample might continue to be degraded after collection. Serum was stored at −20°C until time of analysis. All serum samples were sent on dry ice to a central laboratory where they were stored at −20°C until analysis by HPLC.

### Methods

DAMPA, the catabolic product of CPDG_2_ action on MTX, is known to crossreact with MTX in most commercial immunological MTX assays (Allegra, 1990; Albertioni *et al*, 1996; Widemann *et al*, 1997). Consequently, MTX concentrations determined by commercial laboratories are unreliable following treatment with CPDG_2_. Methotrexate must be assayed by a specific HPLC or enzyme inhibition method.

#### High-pressure liquid chromatography method

Serum MTX was measured by HPLC using a modification of a previously reported method (Brandsteterova *et al*, 1990). After solid phase extraction using C18 cartridges (WatersOasis™ HLB 3 cm^3^, 60 mg (Waters, Eschborn, Germany)), samples were injected onto a 3 *μ*m, Supelcosil™ LC-18-DB 15 cm × 4.6 mm (Sulpelco, München, Germany) radial compression analytical column with a Supelquard™ LC-18-DB 5 *μ*m (Sulpelco, München, Germany) guard column and eluted isocratically with 80 : 20 (v v^−1^) 0.1 mol l^−1^ sodium phosphate (pH 6.8) : methanol (Lichrosolv®) at a flow rate of 1.2 ml min^−1^. Eluent was monitored using a Waters 490 UV adjustable wavelength detector at a wavelength of 310 nm (ThermoQuest, Egelsbach, Germany).

Under these conditions, the retention times for MTX, DAMPA and the internal standard 4-[[2,4-diamino-6-(pteridinyl)methyl]amino]-benzoic acid (Dm-APA) were approximately 5.6, 9.5 and 3.5 min, respectively. The peak areas were evaluated using the Chromquest software (2.5.1, 1998, ThermoQuest, Egelsbach, Germany). Methotrexate and DAMPA were corrected by means of an internal standard in combination with a calibration curve.

### Statistical analysis

Patient data were collected consecutively, recorded on data sheets prepared centrally beforehand, checked by the principal investigator and recorded in a protected database at the University of Bonn. The biometric analysis was performed for all eligible patients who were evaluable (1) for response to the study medication, (2) for the toxicity of CPDG_2_ and (3) for the toxicity of MTX due to complete data documentation. Data were evaluated using descriptive statistical methods (median, ranges, frequencies and percentages).

### Ethics

The study was conducted in accordance with the updated declaration of Helsinki (1996, Somerset West, Republic of South Africa) and approved by the local ethics committees in Bonn, Berlin and Vienna. Prior to enrolment in the study, the patient's parents/legal representatives and/or the patients themselves were informed of the investigational character of the study and the emergency use of CPDG_2_ and had given their written informed consent.

## RESULTS

### Patient characteristics

A total of 82 patients from institutions in Europe were enrolled in this compassionate and emergency use protocol between May 1997 and March 2002. Complete or nearly complete data were available from 65 patients. For 17 patients, no data are available despite repeated telephone and written efforts. The evaluable 65 patients aged 0.9–71.8 years (median 15.4 years) suffered from acute lymphoblastic leukaemia (*n*=26), non-Hodgkin's lymphoma (NHL; *n*=21), osteosarcoma (*n*=12), brain tumours (*n*=3), Hodgkin's lymphoma (*n*=2) and pleural mesothelioma (*n*=1). The patient's characteristics and the different MTX regimens are listed in Table 1. Except for one patient who had clearly increased serum creatinine of 156.6 *μ*mol l^−1^, all patients had an age-dependent normal value of Crea_S_ before treatment (range 17.7–120.4 *μ*mol l^−1^, median 60.2 *μ*mol l^−1^). All patients received intravenous hydration and alkalinisation before, during and after the MTX infusion.

### Contact

Contact with the study centres was established by telephone at a median of 47.5 h (range 19–142 h) after start of the MTX infusion. The MTX levels at the time of contact were between 0.52 and 1082 *μ*M (median 18.0 *μ*M) (*n*=65). Two patients were included in the study with MTX levels of 0.52 *μ*M at 94 h (case 1) and 1 *μ*M at 142 h (case 2) after start of MTX and a serum creatinine of six-fold of the initial value. A total of 12 patients had no documented increase of serum creatinine at the time of contact in contrast to the inclusion criteria. Despite the absent renal failure, the principal investigator (UB) decided to give CPDG_2_ due to very high MTX levels at a late time of contact.

Methotrexate levels and serum creatinine at the first contact and before CPDG_2_ administration are documented in Table 1.

### Carboxypeptidase G_2_ administration

Immediately before CPDG_2_ administration, a median MTX serum level of 11.93 *μ*M (range 0.52–901 *μ*M) was documented (*n*=58).

A single dose of 50 U CPDG_2_ per kg body weight was intended. A vial CPDG_2_ contains 1000 U dry frozen. In all, 57 of 58 evaluable patients received a dose of between 33 and 60 U CPDG_2_ per kg body weight (median 50 U kg^−1^). One patient received only a dose of 17 U CPDG_2_ kg^−1^, although the correct amount was available. Carboxypeptidase G_2_ was administered at a median of 52 h (range 25–178 h) following the start of an MTX infusion of 1.5–12 g m^−2^ over 4–36 h and was well tolerated. At 15 min after CPDG_2_ administration, the MTX serum level of these patients was reduced by around 87%.

Nine patients received a second dose of CPDG_2_. One patient received an additional third dose of CPDG_2_. Six of these nine patients suffered from an osteosarcoma and received an MTX therapy of 12 g m^−2^ over 4 h. An additional three patients suffered from ALL and NHL and received MTX infusion of 5 g m^−2^ 24 h^−1^ (*n*=2) or 8 g m^−2^ 6 h^−1^ (*n*=1), respectively. The median time point of the first CPDG_2_ dose in these patients was 37.5 h after the start of MTX infusions. The second dose was given at a median of 6 h (range 1.5–90 h) after the first dose. One patient received a second dose of CPDG_2_ 1.5 h after the first dose of CPDG_2_ due to logistics.

### Side effects of CPDG_2_

After the administration of CPDG_2_, side effects described by two patients included flushing (*n*=2) and shaking (*n*=1). Both had stable vital signs and the symptoms resolved completely without any intervention.

### Methotrexate levels

**Figure 1:**
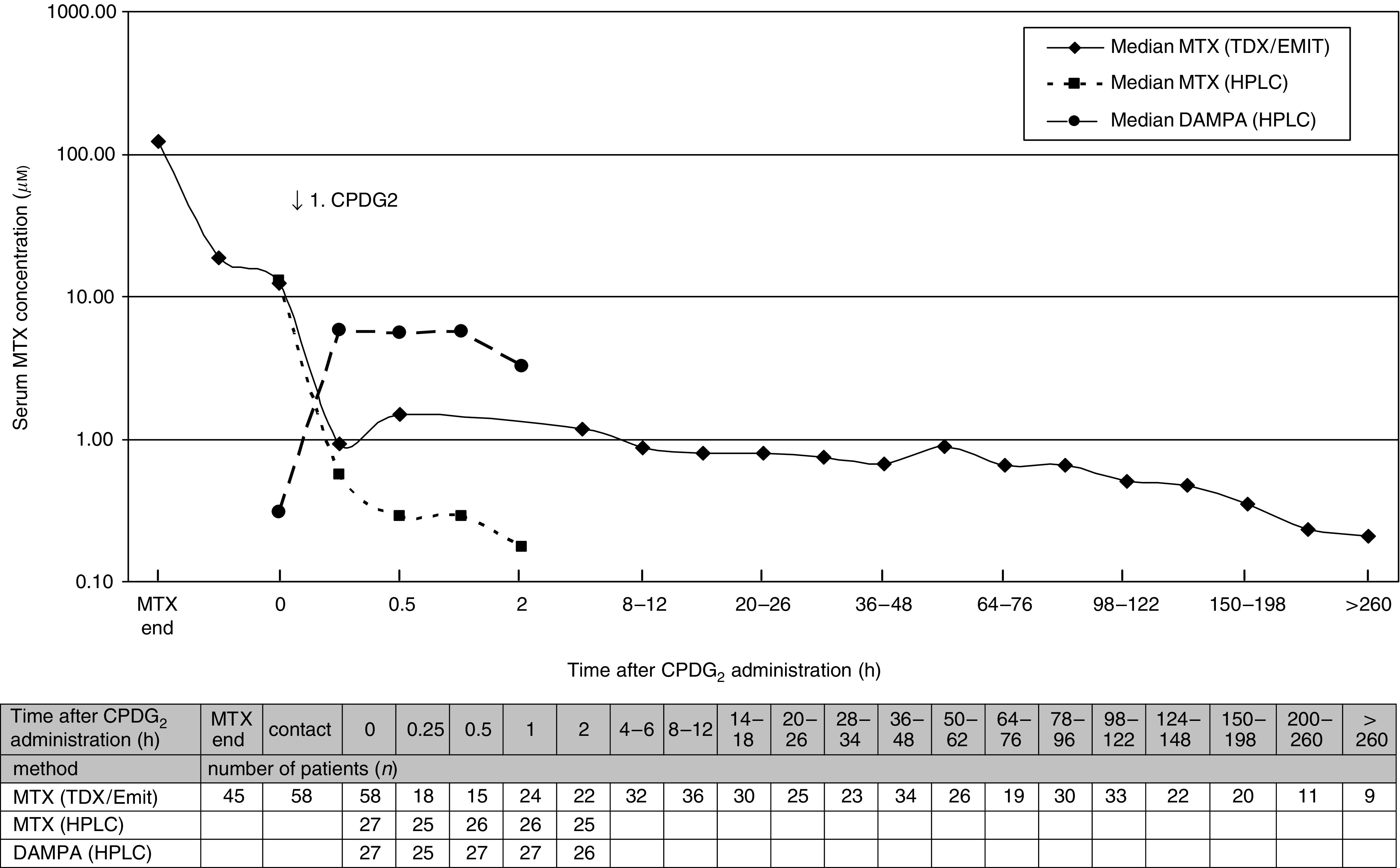
Number and time of MTX samples determined by TDX/EMIT or HPLC and DAMPA samples determined by HPLC after CPDG_2_ injection.

Carboxypeptidase G_2_ resulted in a rapid reduction of serum MTX concentrations in all patients. The MTX concentration determined with immunological MTX detection such as TDX or EMIT showed a median decrease of the MTX level of about 87% (range 70–99%) 15 min after CPDG_2_ administration. Subsequent samples also showed an ongoing decrease of MTX concentrations. Additionally, the MTX concentrations after CPDG_2_ administration were determined by the HPLC method. The MTX levels estimated by HPLC 15 min after the first CPDG_2_ injection decreased by a median of 97% (range 73–99%) (Figure 1). Almost immediately after CPDG_2_ administration, DAMPA was detectable at a concentration comparable to the serum concentration of MTX before CPDG_2_.

Nine patients received an additional dose of CPDG_2_, because their serum MTX concentrations determined by immunological methods remained higher than 1 *μ*M after the first dose of CPDG_2_. The first dose of CPDG_2_ resulted in a median decrease of 88% (Table 2). The second dose of CPDG_2_ did not result in a significant further decline in the serum MTX concentration. Five patients with an MTX level at a median of 8.9 *μ*M (range 3.1–77.6 *μ*M) before the second CPDG_2_ injection showed a second decrease at a median of 26% (range 5–70%). An additional two patients showed an increase after second CPDG_2_ injection of 2 and 26%. In one patient, the MTX level after the second dose of CPDG_2_ is not known and in one case the time between the first and second injections was only 1.5 h and there was no MTX level within the documented level. All patients who received a second dose showed a clearly limited kidney function with median Crea_S_ of 274.4 *μ*mol l^−1^ (range 168.2–654.9 *μ*mol l^−1^) before the second administration.

### Methotrexate-related toxicity (CTC)

In all, 50% of the patients had a reduced Karnowsky index (⩽60%) due to the MTX intoxication. Gastrointestinal complaints with nausea and diarrhoea were frequent. Severe stomatitis (CTC scale ⩾2) occurred in 33% of the patients. A total of 66% of the patients showed an increase of serum creatinine of more than 1.5 times of the initial value before MTX therapy. Increased liver values based on increased GPT/GOT were documented in 58% of the patients with CTC scale ⩾2. Only a small number of patients suffered high-grade toxicity of lung and heart/circulation. In five and six patients, respectively, a mild peripheral and/or central neurotoxicity was observed. Two patients suffered from severe central neurotoxic side effects, a female patient additionally from severe peripheral neurotoxic side effects (CTC scale 4). Further data are listed in Table 3.

### Methotrexate-related mortality

Four patients died due to severe myelosuppression and septic complications 7–22 days following the MTX infusion. Three patients (Table 4, cases 1, 2 and 4) had very high MTX concentrations several hours after the start of MTX, which were lowered by the administration by CPDG_2_ insufficiently. Thus, progressive renal failure and fatal septic complications were observed. In an additional patient (case 3), the MTX concentration was lowered by CPDG_2_ adequately without progressive renal failure. This patient died due to prolonged myelosuppression and its complications (pulmonary haemorrhage due to pulmonary aspergillosis), which were caused by the MTX intoxication and the progressive underlying disease (T-cell ALL) too.

**Figure 2:**
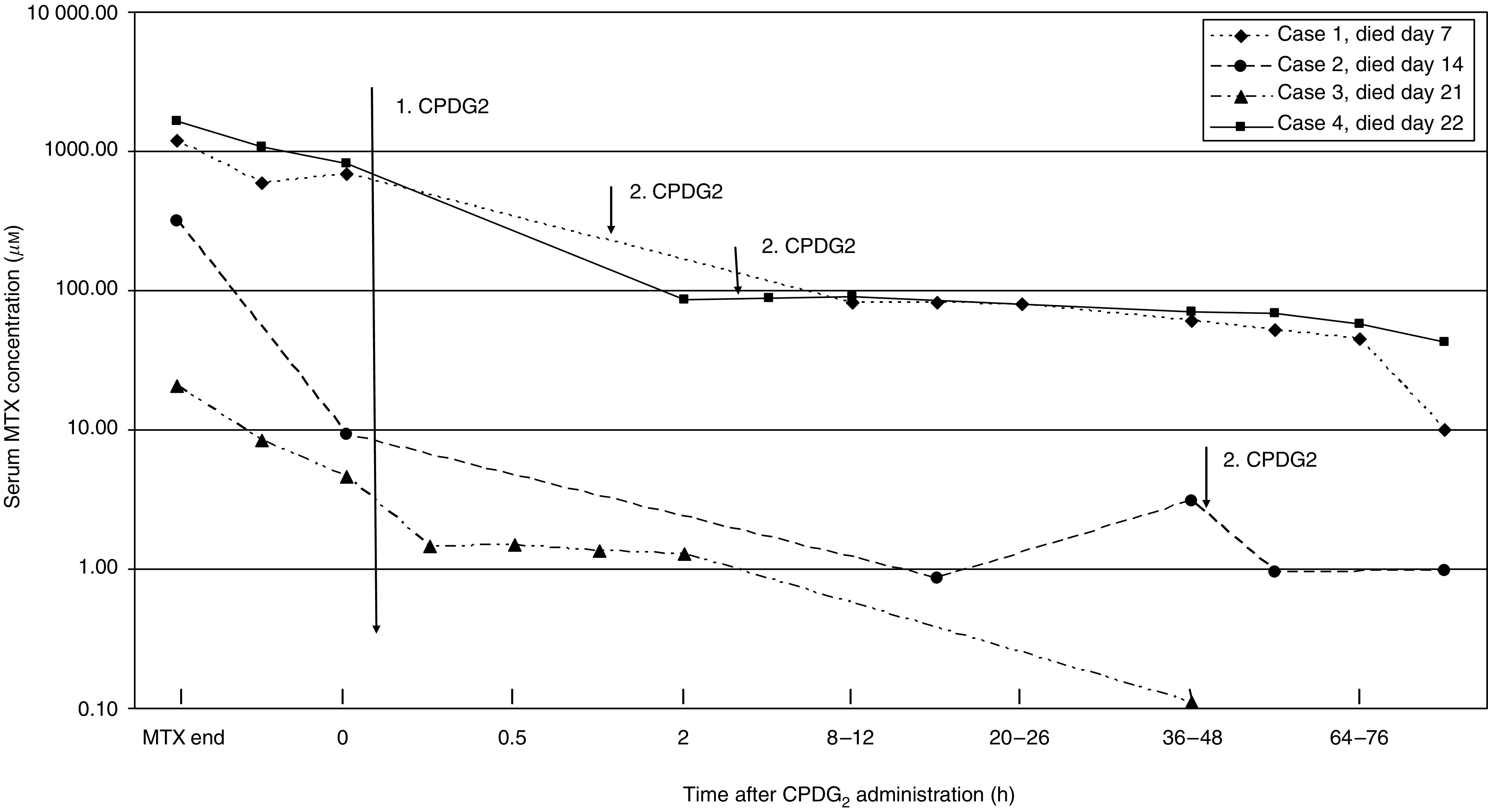
Methotrexate levels after first and second CPDG2 injection in patients who died related to MTX intoxication.

The patient characteristics are shown in Table 4. The course of MTX serum levels is documented in Figure 2.

## DISCUSSION

Methotrexate-induced toxicity is frequently associated with delayed MTX excretion due to renal dysfunction. Delayed MTX excretion leads to prolonged drug exposure and the potential for severe life-threatening toxicity. Patients with impaired renal function prior to the MTX infusion, of advanced age or receiving concomitant nonsteroidal anti-inflammatory drugs or other nephrotoxic substances are at an increased risk of developing renal dysfunction during MTX infusion. In this study, a pre-existing reduced kidney function was known before initiating the MTX therapy in only one patient. All other patients showed increased MTX concentrations unexpectedly without risk factors. Rescue with LV can prevent toxicity but has no effect on delayed MTX excretion.

Carboxypeptidase G_2_ rescue offers the distinct advantage of providing an alternative, rapid route of elimination of MTX by enzymatically catabolising the drug to the inactive metabolite DAMPA.

DAMPA is a minimal inhibitor of dihydrofolate reductase and less soluble than MTX in acidic pH. The faster elimination of DAMPA by comparison with MTX in patients with renal failure, who received CPDG_2_, is due to the extrarenal elimination of DAMPA (Widemann *et al*, 2000). After systemic exposure to CPDG_2_, DAMPA serum concentrations are equivalent to MTX concentrations prior to CPDG_2_ (compare Figure 1). Because of the risk of additional nephrotoxicity of DAMPA, patients must continue to receive fluid hydration and alkalisation.

After the initial dose of CPDG_2_, all patients experienced a rapid (<1 h) and marked (>97%) decrease in serum MTX concentration (determined by HPLC).

In the first years of this study, nine patients received a second dose of CPDG_2_ not resulting in a further marked decrease of the MTX concentration. This could be the result of high DAMPA concentrations inhibiting the hydrolysis of MTX by CPDG_2_ (Widemann *et al*, 1997).

In addition, substantial crossreactivity of the MTX antibodies with DAMPA used in immunoassays results in overestimation of serum MTX concentrations. Therefore, the MTX and DAMPA serum concentration after CPDG_2_ must be monitored by another technique, such as HPLC. The MTX levels estimated by HPLC decreased in median to less than 3% (range 1–27%) of the initial values 15 min after CPDG_2_ injection.

Due to its molecular size, CPDG_2_ is restricted to the extracellular compartment, and the intracellular MTX concentration is initially unaffected by its use. In time, the changed equilibrium between intracellular and extracellular MTX will result in the efflux of intracellular MTX back into the serum, resulting in a rise of serum MTX levels some hours after CPDG_2_ administration, but this is a relatively slow process. For this reason, rescue with LV must be continued following the CPDG_2_ administration.

In this study, six of 65 patients were treated additionally by haemofiltration and/or haemodialysis. These methods must be repeated continuously or daily, in order to reduce the MTX level to nontoxic concentrations. A further risk of these methods results from the necessary vascular entrance, as well as the risk of bleeding secondary to heparinisation and thrombocytopenia. The effectiveness of these procedures cannot be evaluated from our data. Three patients deceased despite these approaches.

In total, four patients died despite CPDG_2_ injection due to severe myelosuppression and septic complications 7–22 days following the MTX infusion. The probable reasons for the lethal complications during a prolonged myelosuppression were (1) very high MTX level with an inadequate decrease of MTX levels after the second CPDG_2_ injection, (2) delayed CPDG_2_ injection (143.5 h after the start of MTX infusion), (3) a progressive underlying disease (T-cell ALL) and (4) inadequate treatment of severe anaemia in a patient who was a Jehovah witness and refused treatment.

In summary, systemic administration of CPDG_2_ followed by long-term LV is highly effective in rescuing patients at high risk of life-threatening MTX toxicity. Carboxypeptidase G_2_ is safe and well tolerated. No severe side effects were observed in this study. Administration of CPDG_2_ is most beneficial for patients with MTX-induced renal failure, especially if it is administered within 48–72 h after the start of MTX infusion.

For the future, it might be helpful to differentiate between patients with extremly high MTX concentrations and severel renal failure and patients with moderately high MTX concentration and renal failure. Since additional injections at CPDG_2_ did not result in a significant further decrease of MTX levels, one initial high dose of 100 U kg^−1^ CPDG_2_ for patients with extremely high MTX concentrations (for example, >100 *μ*mol l^−1^ at 48 h after MTX start and renal failure) may be more effective. For the patients with moderately high MTX levels and renal failure, the application of 50 U kg^−1^ seems to be sufficient. Due to the high costs of the drug, it may be discussed whether an application of 25 U kg^−1^ CPDG_2_ would not be sufficient to lower the MTX levels to nontoxic concentrations in patients with marginal high MTX concentrations and renal failure.

The therapy with CPDG_2_ should always be accompanied by a sufficient hydration, alkalisation and a long-term LV therapy, in order to counterbalance the intracellular MTX. Haemofiltration or haemodialysis might be helpful in oliguric or anuric renal failure. In individual cases with renal failure, severe and lethal courses may occur despite a CPDG_2_ application, in particular if delayed CPDG_2_ administration did not prevent overt toxicity.

## Figures and Tables

**Table 1:** Patient characteristics prior to CPDG_2_ administration

	**Patient characteristics**						**Study entry**		**Prior CPDG_2_ treatment**		**Serum creatinine (*μ*mol l^−1^)**		
**Underlying disease**	**Number of patients (*n*)**	**Sex (female/male)**	**Age (years median range)**	**MTX dose (g m^−2^)**	**MTX infusion time (h)**	**Intrathecal MTX therapy (*n*)**	**MTX serum level (*μ*M) (median (range))**	**Time from start of MTX infusion (h) (median (range))**	**MTX serum level (*μ*M) (median (range))**	**Time from start of MTX infusion (h) (median (range))**	**Prior to MTX infusion (median (range))**	**At study entry (median (range))**	**Prior to CPDG_2_ treatment (median (range))**
All patients	65	24/41	15.4 (0.9–71.8)	1–12	4–36	24	18 (0.52–1082)	47 (19–142)	11.93 (0.52–901)	52 (25–178)	61.1 (17.7–156.6)	185.6 (44.3–516.8)	177.9 (53.1–652.3)
ALL	26	8/18	14.1 (2.6–71.8)	1–8	6 (*n*=1), 24 (*n*=23), 36 (*n*=2)	18	18 (0.52–287)	48 (20–108)	10.66 (0.52–228)	52 (25–120)	45.1 (17.7–115.1)	99.12 (35.4–220.4)	136.3 (53.1–516.8)
NHL/Hodgkin's lymphoma	21/2	9/14	53.3 (4.3–68.9)	3–8	4–8 (*n*=10), 24 (*n*=13)	5	13.94 (1–420)	45 (26–142)	7.46 (0.8–260)	55 (34–178)	70.8 (35.4–144.3)	208.9 (44.3–513.3)	218.6 (44.3–513.3)
Osteosarcoma	12	4/8	15.3 (9.4–39.4)	12	4 (*n*=12)		248 (2.43–1082)	38.5 (19–70.5)	177 (2.43–901)	46 (25–78)	73.5 (35.4–156.6)	172.6 (88.5–516.8)	197.4 (88.5–652.3)
Brain tumour/pleural mesothelioma	3/1	3/1	12.2 (0.9–20.3)	3–5	4 (*n*=1), 24 (*n*=3)	1	3.6 (2.39–17.4)	58 (44.5–66)	2.45 (1.93–17.4)	64.5 (45–82)	63.7 (44.3–97.4)	105.3 (62–380.6)	127.4 (62–407.1)

**Table 2:** Characteristics of patients who got a second CPDG_2_ injection

**Underlying disease**	**Age (years)**	**MTX level prior to first CPDG_2_ (*μ*M)**	**Dose of first CPDG_2_ (U kg^−1^)**	**Time after MTX start (h)**	**MTX decrease (%)**	**MTX level prior to-second CPDG_2_ (*μ*M)**	**Dose of second CPDG_2_ (U kg^−1^)**	**Time after MTX start (h)**	**MTX decrease (%)**	**Last MTX level (*μ*M)**
ALL	17.1	29.7	33	58	77	7.6	33	64	26.32	0.21
NHL	10.2^a^	45	45	143.5	91	3.1	22	192	69.86	3.4
NHL	55	62	47	34.2	84	9.7	47	48.2	8.25	0.07
Histiocytoma	39.4^a^	688	54	37.5	88	NA	54	39	NA	8.06
Osteosarcoma	15.4	306	46	63.4	74	13.4	46	84.4	(−) 26.12^b^	3.4
Osteosarcoma	27.9	138	36	34.5	94	8.9	55	124.5	43.82	0.1
Osteosarcoma	9.4	596	50	32	84	77.6	20	38	4.64	0.83
Osteosarcoma	19.1^a^	815	47	24.8	89	87.5	47	28.5	(−) 2.29^b^	25.49
Osteosarcoma	14.4	901	45	50.8	90	1.26	45	56	NA	NA

a Patient died.

b Negative values are due to a rerise of the MTX concentration.

NA=not available.

**Table 3:** Methotrexate-related toxicity

		**CTC scale (number of patients (*n*) (%))**				
**Kind of toxicity**	**Number of evaluable patients (*n*)**	**0**	**1**	**2**	**3**	**4**
Infection	40	27 (67.5)	1 (2.5)	7 (17.5)	1 (2.5)	4 (10.0)
Fever	38	26 (68.4)	3 (7.9)	8 (21.1)	1 (2.6)	0 (0)
Gastrointestinal						
Vomiting	43	13 (30.2)	11 (25.6)	8 (18.6)	7 (16.3)	4 (9.3)
Nausea	39	12 (30.8)	11 (28.2)	7 (17.9)	7 (17.9)	2 (5.1)
Oral mucositis	39	17 (43.6)	9 (23.1)	7 (17.9)	6 (15.4)	0 (0)
Diarrhoea	41	24 (58.5)	9 (22.0)	5 (12.2)	1 (2.4)	2 (4.9)
Skin	42	29 (69.0)	9 (21.4)	4 (9.5)	0 (0)	0 (0)
Haemorrhage	39	32 (82.1)	2 (5.1)	2 (5.1)	0 (0)	3 (7.7)
Kidney						
Creatinine	41	7 (17.1)	7 (17.1)	13 (31.7)	9 (22.0)	5 (12.2)
Creatinine clearance	25	6 (24.0)	5 (20.0)	4 (16.0)	5 (20.0)	5 (20.0)
Proteinuria	31	21 (67.7)	5 (16.1)	3 (9.7)	1 (3.2)	1 (3.2)
Haematuria	34	23 (67.6)	7 (20.6)	3 (8.8)	0 (0)	1 (2.9)
Liver						
GOT/GPT	40	12 (30.0)	5 (12.5)	10 (25.0)	6 (15.0)	7 (17.5)
Bilirubin	40	20 (50)	12 (0)	7 (17.5)	0 (0)	1 (2.5)
Liver clinically	27	24 (88.9)	0 (0)	2 (7.4)	0 (0)	1 (3.7)
Lung	40	33 (82.5)	0 (0)	1 (2.5)	3 (7.5)	3 (7.5)
Heart/circulation						
Cardiomyopathy	40	36 (90.0)	0 (0)	1 (2.5)	0 (0)	3 (7.5)
Echocardiography	19	18 (94.7)	0 (0)	0 (0)	0 (0)	1 (5.3)
Neurotoxicity						
Central	38	31 (81.6)	5 (13.2)	0 (0)	0 (0)	2 (5.3)
Peripheral	38	31 (81.6)	6 (15.8)	0 (0)	0 (0)	1 (2.6)

**Table 4:** Characteristics of patients who died related to MTX intoxication

**Case**	**1**	**2**	**3**	**4**
Diagnosis	Histiocytoma	NHL	ALL	Osteosarcoma
Age (years)	39.4	10.2	5.7	19.1
Time of death (days after MTX start)	7	14	21	22
Causes of death	Septic, multiorgan failure	Septic, multiorgan failure	Septic, multiorgan failure, progressive underlying disease, haemorrhage due to pulmonary aspergillosis	Cardiopulmonal failure due to severe anaemia, being Jehovah witness
MTX therapy	12 g m^−2^ 4 h^−1^	5 g m^−2^ 24 h^−1^	1 g m^−2^ 36 h^−1^	12 g m^−2^ 4 h^−1^
Contact after MXT start (h)	32	140	49.5	19
MTX level prior to first CPDG_2_ (*μ*M)	688	9.2	4.58	814.6
Creatinine concentration prior to first CPDG_2_ administration (*μ*mol l^−1^)	194.7	150.5	69	327.5
Maximum creatinine level (*μ*mol l^−1^)	230.1	416	85.8	567.3
Dose of first CPDG_2_ (U kg^−1^)	54	45	50	47
Time after MTX start (h)	37.5	143.5	50	25
MTX level (*μ*M)/MTX decrease (%) after CPDG_2_	82.8/88	0.85/91	1.46/86	86.1/90
MTX level prior to second CPDG_2_ (*μ*M)	NA	3.1		86.1
Dose of second CPDG_2_ (U kg^−1^)	54	22		47
Time of second CPDG_2_ after MTX start (h)	39	192		28.5
MTX level (*μ*M)/MTX decrease^a^ (%) after second CPDG_2_	NA	0.94/70		87.5/−1.6^a^
Leucovorin rescue	Adequately	Not known	Adequately	Adequately
Haemodialyse/haemofiltration (days)	3	8		5
Start of haemodialysis after MTX infusion (h)	55	171		24
Severe myelosuppression	No	Yes	Yes	Yes

a Negative value is due to a rerise of the MTX concentration. NA=not available.
